# Regeneration in Echinoderms: Molecular Advancements

**DOI:** 10.3389/fcell.2021.768641

**Published:** 2021-12-17

**Authors:** Joshua G. Medina-Feliciano, José E. García-Arrarás

**Affiliations:** University of Puerto Rico, Río Piedras Campus, Río Piedras, Puerto Rico

**Keywords:** echinoderm, regeneration, gene function, transcriptome analyses, signaling pathways, dedifferentiation

## Abstract

Which genes and gene signaling pathways mediate regenerative processes? In recent years, multiple studies, using a variety of animal models, have aimed to answer this question. Some answers have been obtained from transcriptomic and genomic studies where possible gene and gene pathway candidates thought to be involved in tissue and organ regeneration have been identified. Several of these studies have been done in echinoderms, an animal group that forms part of the deuterostomes along with vertebrates. Echinoderms, with their outstanding regenerative abilities, can provide important insights into the molecular basis of regeneration. Here we review the available data to determine the genes and signaling pathways that have been proposed to be involved in regenerative processes. Our analyses provide a curated list of genes and gene signaling pathways and match them with the different cellular processes of the regenerative response. In this way, the molecular basis of echinoderm regenerative potential is revealed, and is available for comparisons with other animal taxa.

## Introduction

Regeneration is a phenomenon present, to some degree, in all metazoans from sponges to vertebrates. However, the extent of an organism’s regenerative properties can vary significantly within a taxonomic group*.* In general terms, animals in deuterostome clades that radiated before vertebrates show great regenerative capabilities (Bely and Nyberg, 2010). Among the animal groups that are closely related to the chordates (e.g., humans), those in the Echinodermata phylum encompass some of the most advanced regenerative species. As deuterostome invertebrates they have been extensively used as model species, mainly because of the facility to perform developmental and molecular studies that have provided important information to development and molecular biology fields (Gahn and Baumiller, 2010). In the last decades, echinoderms have been slowly gaining special attention as model systems for regeneration studies due to their wide assortment of astonishing regenerative capacities (Hyman, 1955; Candia Carnevali and Bonasoro, 2001). Their use to probe the molecular underpinnings of regenerative processes and the possibility of comparative studies with other deuterostomes, including chordates, promise to shed some light into one of the oldest questions in Regenerative Biology: Why can some animals regenerate organs and body parts while others lack this ability?

**FIGURE 1:**
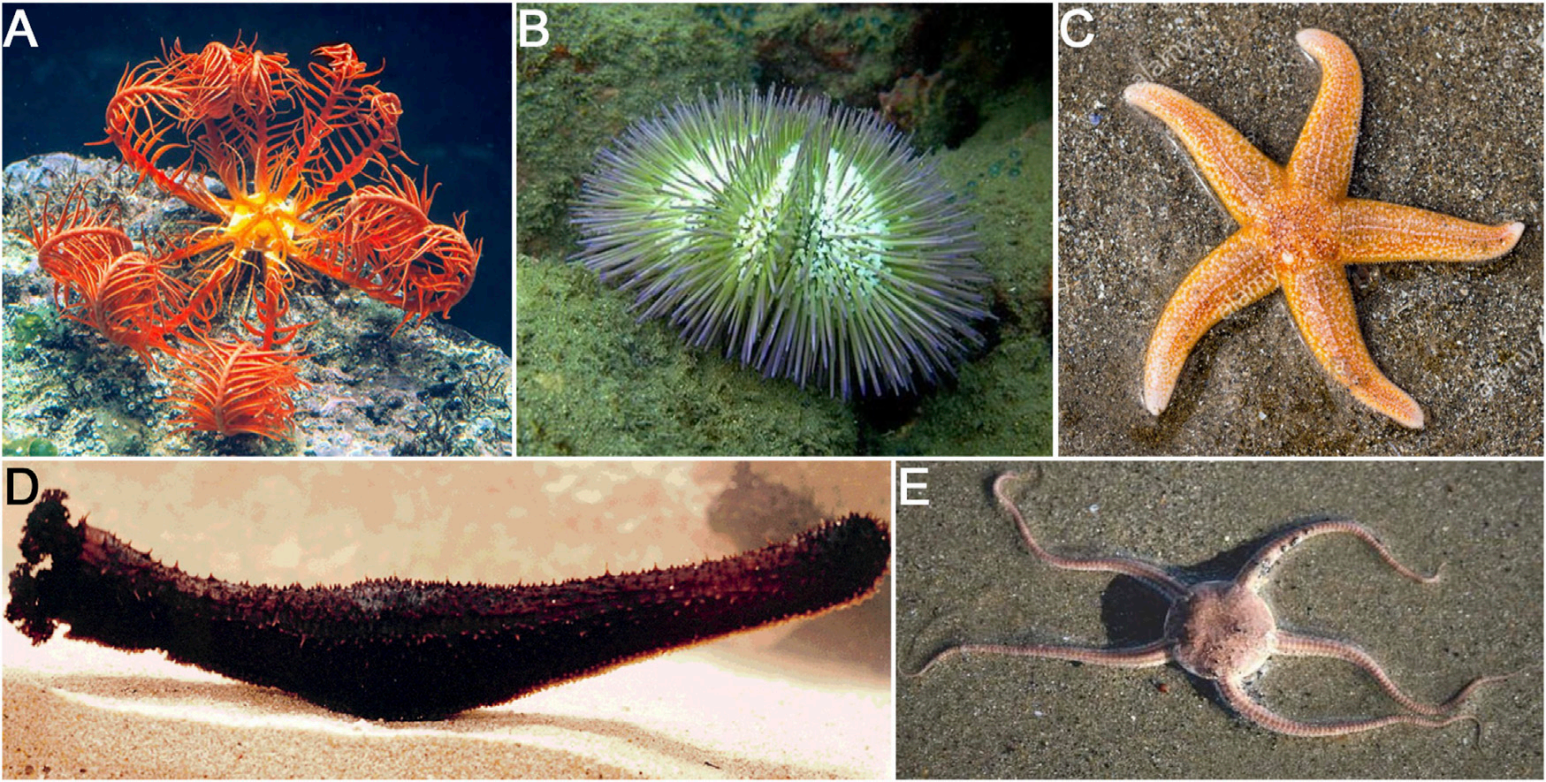
Representative of the five classes in the Echinodermata phylum that have been used in regeneration studies. **(A)** Crinoidea represented by the feather star *Antedon mediterranea*. **(B)** Echinoidea represented by the sea urchin *Lytechinus variegatus*. **(C)** Asteroidea represented by the sea star *Asterias rubens*. **(D)** Ophiuroidea represented by the brittle star *Amphiura filiformis*. **(E)** Holothuroidea represented by the sea cucumber *Holothuria glaberrima.*

The phylum Echinodermata is composed of five major classes: Crinoidea (feather stars), Asteroidea (sea stars), Echinoidea (sea urchins), Ophiuroidea (brittle stars), and Holothuroidea (sea cucumbers) (Figure 1). The degree of regenerative competences of echinoderms varies among the different classes. Sea urchins exhibit the lowest regenerative capacity but still can regenerate parts of its their test, broken or lost spines and pedicellariae (Dubois and Ameye, 2001). Brittle stars are well known for their arm regeneration prowess following amputation (Dupont and Thorndyke, 2006). Sea stars are also able to regenerate arms and pedicellaria, are capable of regenerating their pyloric caeca (Anderson, 1962; Anderson, 1965), and in some cases can regrow complete organisms from remnant arms (Wilkie, 2001; Ducati et al., 2004; Ben Khadra et al., 2018). Similarly, crinoids are also capable of regenerating arms as well as whole crowns and viscera (Amemiya and Oji, 1992; Candia Carnevali and Bonasoro, 2001; Wilkie, 2001; Kondo and Akasaka, 2010; Ben Khadra et al., 2018). However, it is within the holothuroid class that multiple regeneration processes have been documented. These animals are known to undergo regeneration of various organs, including respiratory trees, longitudinal muscles, radial nerve cord, tentacles, polian vesicles, and digestive tract, among others. The regenerative capacities of holothurians extend even further, with species known to regenerate full organisms after fission and even from remnant body parts (Smith, 1971; Kamenev et al., 2013; García-Arrarás et al., 2018).

In addition to the studies of adult echinoderms described above, several investigators have focused their research on regeneration processes of echinderm larvae (Cary et al., 2019). It has been documented that echinoderm larvae can regenerate certain structures and are even able to undergo fission and clone themselves to produce two embryos from the original one (Vickery et al., 2001; Eaves and Palmer, 2003). More recently, (Kasahara et al., 2019), showed that the larvae of two sea urchin species are able to regenerate the “cell mass” responsible for adult rudiments when removed. These and other studies underscore the impressive regeneration capacities of larval echinoderms, opening the possibility of using larvae to probe molecular mechanisms of the regeneration processes. In this respect it is important to highlight the rich literature of echinoderm developmental processes, both at the cellular and molecular level. In fact, the development of the sea urchin embryo is probably the best-studied model system showing the gene regulatory network behind the formation of embryonic structures from the fertilized egg. Thus, the combination of what is known from embryonic developmental studies and regeneration studies promises to provide important information to explain the molecular basis of echinoderm regenerative processes. While the present review focuses on adult echinoderms, readers interested in larval regeneration are directed to a recent review (Wolff and Hinman, 2021) on the regeneration of sea star and sea urchin larvae that collects most of the information available on this topic.

Certainly, the phylum Echinodermata contains some of the most suitable species to perform in depth studies in Regenerative Biology. Here we revise the echinoderm species that have been used as models to study the molecular basis of the regeneration of different tissues and organs, and the molecular findings that have been generated from these studies. It is important to highlight that while there are numerous echinoderm molecular studies, here we only focus on those directed to their regeneration potential. Therefore, an extensive section of the article focuses on ophiuroids and holothuroids, being the only species, whose regeneration has been studied from *in situ* characterization up to functional studies. Complementary to this revision we would like to direct the readers to two previous reviews on the topic that provide some of the first insights into this area of research (Thorndyke et al., 2001; Mashanov and García-Arrarás, 2011) and to a recent comprehensive review by Dolmatov (2021) on the molecular aspects of regeneration mechanisms in holothurians.

## Molecular Studies of Regeneration

Molecular studies of echinoderm regeneration can be grouped into three different categories. The first category includes those studies limited to individual genes and focuses on the presence and/or expression of one or a few genes at a time. These studies provide for the study of candidate genes, in particular those that have been previously associated with regenerative or developmental processes, to be searched in the regenerating tissues of echinoderms. This is usually done by determining the gene’s mRNA or protein product within the regenerating structure. In some cases, these studies include some type of quantification of expression between regenerating structures and the normal non-injured organ. The evidence in these studies is of the sort of “guilt by association” where the presence of the gene product by itself is assumed as evidence for a possible role in the regeneration process.

The second category groups those studies where multiple genes are analyzed with some high-throughput method, mainly microarrays or RNA sequencing technology. In these studies, gene expression is quantified to determine differential gene expression levels between the regenerating structure and the normal (uninjured) tissue or organ. Differential expression provides evidence that might suggest specific genes for a role in the regenerative processes and might also serve for the identification of novel genes in the processes. Although the results are correlative and do not provide for functional relationship between the identified genes and the regeneration process, the identification of specific genes can lead to further functionality studies.

The third category comprises the studies where the functions of the candidate genes are tested. Two main strategies have been used to study functionality: 1) pharmacological experiments where drugs are used to activate or inhibit a gene product or its associated signaling pathway(s), and 2) gene knockdowns using RNAi technology to decrease the expression of a gene of interest. These are promising strategies that are in continuous optimization for echinoderm models in order to advance to levels similar to those of other regeneration models.

**FIGURE 2:**
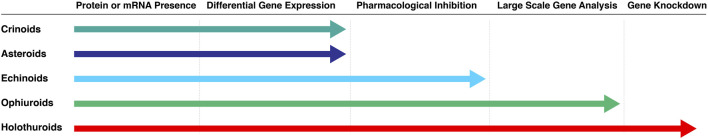
Molecular studies of regeneration favor certain echinoderm classes over others. Extensive studies have been done in ophiuroids and holothuroids. Asteroids and crinoids have been less studied at the molecular level, even though their regeneration prowess is well known, and histological and cellular analyses of their arm regeneration are available. Molecular studies in echinoids are also limited, mainly because their regenerative capabilities are the least impressive when compared to those from other classes.

Not all echinoderm classes have been studied to the same depth at the molecular level. As shown on Figure 2, certain groups have barely been studied while studies in others are farther advanced (i.e., ophiuroids and holothuroids). The following sections provide a more extensive review of the molecular studies within the three categories.

### Category 1- Individual Genes

Regeneration-associated genes have been identified in all echinoderm classes. *In situ* hybridization and immunohistochemical techniques have been the principal techniques to localize the expression of the mRNA or protein for the genes of interest, while qRT-PCR has been used to quantify their expression. In crinoids, a few studies have addressed putative genes that might be associated to arm regeneration. Among the most important are the experiments by (Patruno et al., 2002; Patruno et al., 2003) documenting the expression of putative members of the Transforming Growth Factor (TGF) and Bone Morphogenetic Protein (BMP) families in cells of the regenerating arm of the crinoid *Antedon bifida*. An increase in the expression of the molecules at certain regeneration stages was associated with their possible involvement with cellular regenerative events, particularly with cellular migration. These initial experiments have shed light on the molecular basis of crinoid regeneration. Nonetheless, it is daunting the lack of high throughput experiments or at least multiple gene comparisons in an animal with outstanding regenerative capabilities, and one that holds a key position at the Echinodermata phylum. Therefore, this void serves as a reminder of the opportunities available for those that wish to focus on echinoderm regenerative biology.

There are also very limited studies on the molecular regeneration in sea urchins, and most of these are also focused on the presence and differential expression of single genes associated with the regeneration of spines or pedicellaria (Dubois & Ameye, 2001). Therein, the expressions of Notch target genes and of stem cell associated genes, *Piwi*, *Vasa*, and *tert* were analyzed during spine and tube feet regeneration in *Lytechinus variegatus* (Reinardy et al., 2015; Bodnar and Coffman, 2016). Similarly, genes associated with mineralization have been studied in experiments aimed at determining a possible effect of ocean acidification on spine regeneration (Emerson et al., 2017). Surprisingly, the species *Strongylocentrotus purpuratus* has never been the subject of regeneration studies, even though its genome was among the first to be sequenced and has served as the groundwork for molecular studies in many other species.

Among ophiuroids, the brittle star *Amphiura filiformis* has been the main species for studies on specific genes associated with regenerative processes, particularly arm regeneration. In one of the earlier studies, *Afuni*, a member of the BMP family, was shown to be expressed in *A. filiformis* regenerating arm (Bannister et al., 2005). This initial finding was followed by a second member of the BMP family (*BMP2/4*), also shown to be highly expressed in the regenerating arms of the same species (Bannister et al., 2008). Similarly, *Hox* gene sequences were identified from mRNA of brittle stars regenerating arm tips (Ben Khadra et al., 2014). Subsequent studies expanded the number of genes studied to include several transcription factors (*alx1*, *ets1/2*, *foxN2/3*, *gataC*, *nk7*, *soxE*, and *twist*) associated with skeletal and muscle tissues (Czarkwiani et al., 2013) and immune system related genes (Ferrario et al., 2018). Transcription factors have become key targets in recent studies, being crucial for processes intrinsic to regeneration such as cellular differentiation. Other studies focused on the presence (or absence) of extracellular matrix (ECM) genes (Ferrario et al., 2020), skeletogenic genes (Piovani et al., 2021) and glucosaminoglycans (Ramachandra et al., 2014; Ramachandra et al., 2017) as possible mediators of the arm regeneration process.

In holothurians, the initial studies addressing the molecular basis of intestinal regeneration were directed to the determination of changes in gene expressions. One of the initial studies done in *Holothuria glaberrima* led to the identification of the presence of the first *serum amyloid A* (*SAA*) ortholog in an invertebrate deuterostome (Santiago et al., 2000). Through further analyses, using northern blot and immunohistochemistry techniques, SAA was found to be highly expressed in the coelomic epithelia during the mid-late stage of intestinal regeneration. Likewise, using similar techniques, another study in *H. glaberrima* demonstrated for the first time the presence of an ependymin-related gene, long thought to be a vertebrate-specific gene. The expression of this gene was analyzed utilizing RT-PCR, demonstrating high expression around the first week of regeneration, when the initial intestinal rudiment is being formed (Suárez-Castillo et al., 2004). Other experiments using *in situ* hybridization documented the expression of various genes in the regenerating intestinal rudiment, including *survivin*, *mortalin*, *Wnt9*, *TCTP*, and *BMP/Tll* (Mashanov et al., 2010; Mashanov et al., 2012a). Furthermore, one of the initial attempts to characterize gene expression changes at a larger scale was done by analyzing cDNA libraries of regenerating animals through differential library screening (Rojas-Cartagena et al., 2007).

### Category 2—Arrays and Transcriptomes

As modern sequencing technologies continue to develop, major advancements have been achieved towards uncovering the underpinnings of regenerative processes. These applications have allowed scientists to divert from studying only candidate genes to perform large-scale molecular studies. Certainly, the sequencing of the first echinoderm genome, that of the purple sea urchin *S. purpuratus,* followed by that of the sea star *Patiria miniata* and the sea cucumber *Apostichopus japonicus* set the groundwork for numerous molecular studies in echinoderm species (Sea Urchin Genome Sequencing Consortium, 2006; Kudtarkar and Cameron, 2017; Zhang et al., 2017). These high-throughput analyses are necessary to visualize the expression trends of all the key factors involved in the regenerative process. Ophiuroids and holothurians are the most used echinoderms in large-scale sequencing studies (Tables 1, 2). Specifically, regeneration has been mainly studied at this level in six species: the brittle stars *A. filiformis, Ophionotus victoriae*, and *Ophioderma brevispina,* where the focus is on arm regeneration and the sea cucumbers *Eupentacta fraudatrix*, *A. japonicus*, and *H. glaberrima* as models of intestinal regeneration. Regeneration of the radial nerve has also been studied in *H. glaberrima*.

**TABLE 1:** Transcriptome profiling of regenerating tissues in brittle stars.

Species	Tissue	Stage	Method	References
*A. filiformis*	Regenerating arm	Differentiation stages: blastema-like formation, 50% differentiation, and 95% differentiation	Microarray	Burns et al. (2011)
	Regenerating arm explants	7 dpa	Microarray	Burns et al. (2012)
	Regenerating arm	1 and 3 dpa	Illumina RNA-seq and Proteomic analyses	Purushothaman et al. (2015)
*O. victoriae*	Regenerating arm	Pooled weekly for 4 weeks and monthly during 12-months post amputation	454 pyrosequencing RNA-seq	Burns et al. (2013)
*O. brevispina*	DAPT-treated regenerating arms	14 dpa	Illumina RNA-seq	Mashanov et al. (2020)

In this study tissue was compared to normal mesentery.

**TABLE 2:** Transcriptome profiling of regenerating tissues in sea cucumbers.

Species	Tissue	Stages	Method	References
*H. glaberrima*	Regenerating intestine	3-dpe and 7-dpe	EST	Rojas-Cartagena et al. (2007)
	Regenerating intestine	3-dpe, 7-dpe, 14-dpe	Microarray	Ortiz-Pineda et al. (2009)
	Regenerating intestine	1-dpe vs. 3-dpe	Illumina RNA-seq	Quispe-Parra et al., (2021a)
	Regenerating radial nerve cord	2, 12, 20 days post injury	454 pyrosequencing RNA-seq	Mashanov et al. (2014)
*A. japonicus*	Regenerating intestine/body wall	Pooled body wall (4 days of regeneration) and intestine (7-dpe)	454 pyrosequencing RNA-seq	Sun et al. (2011)
	Regenerating intestine	3-,7-, 14-, 21-dpe	Illumina RNA-seq	Sun et al. (2013)
	Regenerating intestine	0.5, 2, 6 h, 3-dpe, 5-dpe, 7-dpe, 14- dpe, and 21-dpe	Illumina RNA-seq	Zhang et al. (2017)
*E. fraudatrix*	Regenerating intestine	3-, 5-7-, 10–12-dpe	Illumina RNA-seq	Boyko et al. (2020)

In this study tissue was compared to normal mesentery.

#### Brittle Star Regeneration

To date, various reports have been done that employ large-scale molecular techniques to study regeneration in the ophiuroid *A. filiformis* (Table 1)*.* These studies have focused on arm regeneration, a process that has been well-described at the histological and cellular level (Biressi et al., 2010; Czarkwiani et al., 2016; Ferrario et al., 2018, Figure 3A). In brief, soon after severing the arm, the injured tip is healed and re-epithelized. This is followed by the formation of what the authors refer to as a blastema [recent work questioned whether it is a real blastema (Czarkwiani et al., 2016), therefore, we will continue to use the term blaster-like structure to describe it]. The formation of this blastema-like structure signals the beginning of the regenerative process. As the bud grows, new tissues and organs such as the water vascular canals and the radial nerve, reappear, and eventually the first new arm segment is defined. Subsequent regeneration leads to the formation of new segments at the distal tip position. Although there are differences in the design and focus of each project (e.g., the time of sample collection after amputation/autotomy and the specific amputation site), similar gene-associated processes stand out in all the analyses. Compiling the findings of these studies, the most differentially expressed genes can be grouped in a few categories, such as developmental, ECM-related, and cytoskeleton genes. Also, they are mainly classified to be part of transcription, translation, cellular, development, and metabolic processes.

**FIGURE 3:**
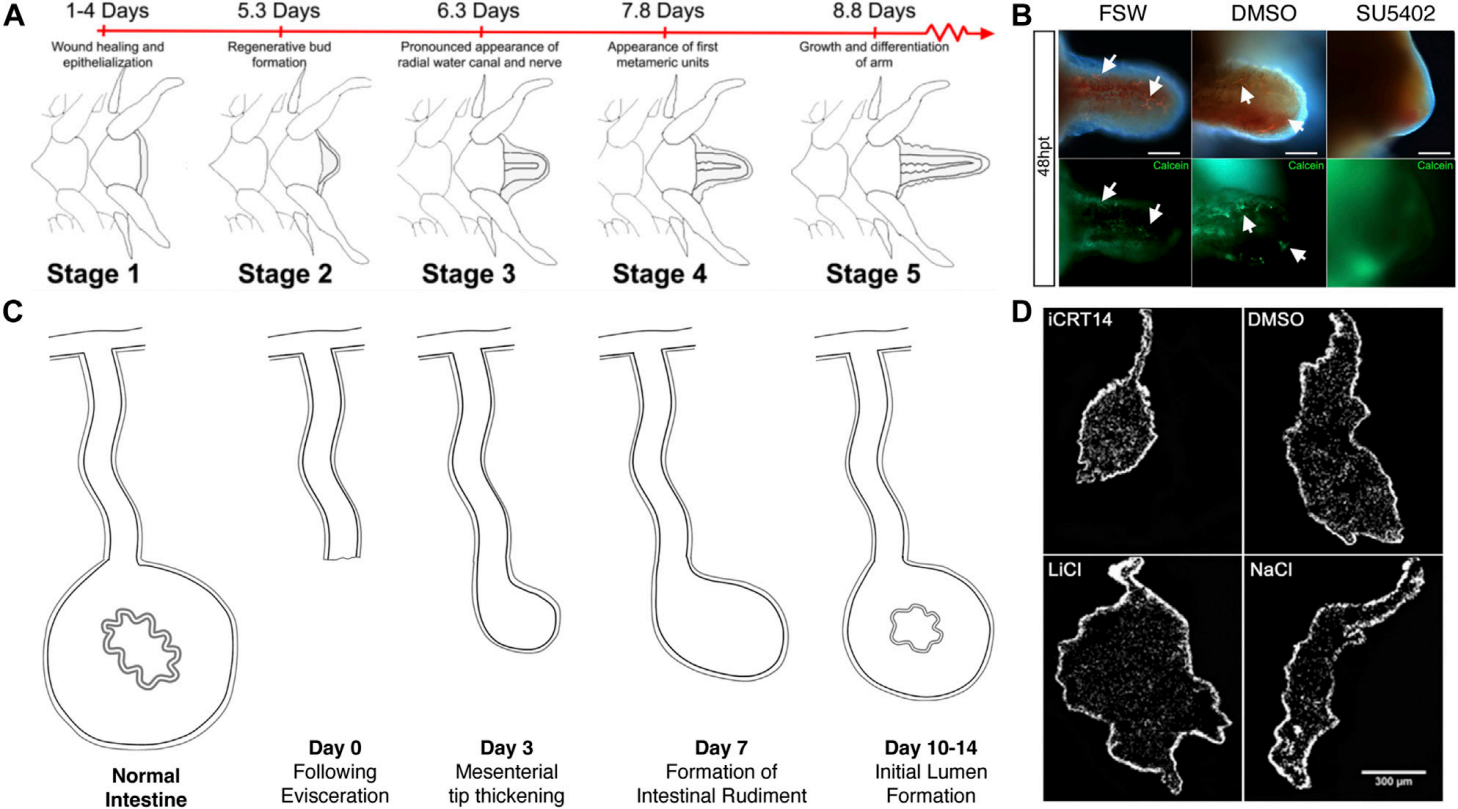
Pharmacological studies in brittle star arm regeneration and sea cucumber intestinal regeneration. **(A)** Stages of arm regeneration in the brittle star *A. filif*ormis provide a baseline to detect the effect of regeneration modulating drugs as shown in Czarkwiani et al. (2016). **(B)** The effect of a FGF inhibitor in a study by Czarkwiani et al. (2021) is shown by a decrease in the extension of the regenerating arm and by the inhibition of formation of the spicules that form the skeleton. **(C)** Stages of intestinal regeneration in the sea cucumber *H. glaberrima* provide a baseline to detect the effect of regeneration modulating drugs as shown in García-Arrarás et al., 2019. **(D)** Results from Bello et al. (2020) on the effect of Wnt inhibitors (iCRT14) and activators (LiCl) is determined in the size of the regenerating gut rudiment when compared to those of vehicle treated controls.

The earliest high-throughput molecular study of *A. filiformis* used cDNA microarrays to search for genes differentially expressed in regenerating arms (Burns et al., 2011). They studied the three stages previously defined by a key differentiation patterns guide (Dupont and Thorndyke, 2006) to identify 4,072 genes with significant differential expression between the selected stages compared to non-regenerating arms. Among the stages, the first and second stages (the blastema-like formation and 50% differentiation stage, respectively) shared a high number of differentially expressed genes (488 upregulated and 743 downregulated). The first stage contained the highest percentage of differentially expressed genes, which suggested a higher transcription activity due to the numerous events required to form the blastema-like structure. At this stage, high expression of genes associated with transcription, translation, and cell energy, including cytochrome oxidase, members of the solute carrier, elongation factors, and ribosomal proteins were found. Other highly expressed genes identified were associated to cell proliferation, division, and apoptosis (e.g., cleavage stimulation factor, polyubiquitin, proteasome, actin, and collagen). Among the developmental genes identified as upregulated in early regeneration were the *high mobility group box 1* (*HMGB1*-known to be involved in *Hox* regulation), *hyalin*, and *Sox1*. Other developmental genes commonly associated with regeneration, such as *Hox* and *Wnt*, were not found at any stage. The expression of the developmental genes upregulated at the first stage was different from those upregulated during the third stage, as would be expected due to the occurrence of different cellular events. Specifically, at the third stage the expression of *hyalin* and *Sox1* genes decreased, while BMP*-1* homolog, which was downregulated at the first stage, increased its expression to normal levels.

This initial microarray study by Burns et al. (2011) was followed by a report using 7-days arm regenerating explants *in vitro* (Burns et al., 2012). These explants were shown to continue their regeneration process in culture, and they were used to assess transcription profiles using microarrays. Three regions of the arm explant were analyzed: the proximal, medial, and distal portions (in relation to the site of arm amputation). After the initial arm autotomy, a second amputation was made at the tip of the arm (the distal portion), and as expected, most of the transcription activity was localized to that distal region (which eventually forms the blastema-like structure). They identified a total of 1,733 differentially expressed transcripts among all samples, from which 791 were sequenced. As expected, the identified genes were similar to those found in their previous *in vivo* study of 7-days regenerating blastema-like structure (Burns et al., 2011). The main changes were in expression levels rather than in the presence or absence of gene expression. Developmental genes, such as *Notch1* and *Sox1,* were also upregulated as shown before*.* Other genes upregulated in the *in vitro* regenerating explants, are also involved with proliferation, migration, and differentiation, such as *frizzled*, *tetraspanin*, and *selenoprotein W,* respectively*.* From these, *selenoprotein W* gene was highly expressed at the second stage of Burns et al. (2011) study. Strikingly, this gene has been shown to be highly expressed in myoblasts proliferation (Loflin et al., 2006), which is in concordance with previous reports suggesting it is associated with the myocyte differentiation that takes place in early regeneration stages of *A. filiformis* (Biressi et al., 2010). While upregulation of *frizzled* was detected, none of its ligand *Wnt* molecules were found to be upregulated. Furthermore, *DSP-1,* another development-related gene involved in *Hox* regulation, was also found to be upregulated in the distal portion of the arm explant. Beyond genes associated to developmental processes, there were additional genes and transcription factors related with other components and processes as we will see below.

An additional report used a different approach to study the transcriptome of *A. filiformis* regenerating arm *in vivo* (Purushothaman et al., 2015). They focused on earlier stages [1- and 3-days post-amputation (dpa)] prior to the blastema-like structure formation stage when tissue repair processes are known to occur (Biressi et al., 2010). In this study, RNA sequencing (RNA-seq) was performed along with proteomic analyses, providing a higher level of confidence and confirmation of the molecular acitivtiy during this process. Despite the sampling difference, the results strongly correlate with the reports of Burns et al. (2011), Burns et al. (2012). Out of the assembled contigs, 694 annotated genes and 194 proteins were identified as differentially expressed. Among significant genes with highest expression were craniofacial development protein 1 (*CFDP2*), THO complex (*THOC1*), transitional endoplasmic reticulum atpase (*TER94*) and adenosine kinase (*adk*). In contrast, transforming growth factor beta-2 (*Tgfb2*), AT-rich interaction domain 1b (*ARID1b*), SIPA, and N-ethylmaleimide-sensitive fusion protein (NSF) were at the top of the downregulated transcripts list. Furthermore, by correlating differential expression profiles of genes and protein products at 1- and 3-dpa, it was possible to identify pathways and processes potentially involved in the regeneration process. Important processes identified through Gene Ontology (GO) analysis included: metabolic, catabolic, translation initiation, and elongation. They also identified genes involved with the cytoskeleton and ECM, such as actin and collagen genes. Nevertheless, compared to previous studies, their focus was towards identifying genes associated with regeneration-related pathways. Among these there were the development-related vascular endothelial growth factor (VEGF) pathway and cytoskeleton remodeling pathways. Genes from these pathways demonstrated dynamic expression patterns. For instance, the VEGF pathway downregulated genes were Protein kinase C (*PKC*), extracellular protein kinase (*ERK1/2*) and ERK1 (MAPK3). Comparatively, *AKT,* Actin cytoskeleton transcripts, and I-kB were upregulated. Among the identified genes from the cytoskeleton remodeling pathway, alpha-actin one and multiple eukaryotic initiation factors (eIF; i.e., eIF4G1, eIF4G3) appeared as downregulated. In comparison, eIF4G2, MSK1, myosin light chain phosphatase (*MLCP*) and Actin cytoskeleton transcripts showed upregulation. Other identified pathways showed involvement of ECM and cytoskeleton related genes, such as the Integrin mediated cell adhesion and migration pathway. Noteworthy, they also identified the translation regulation pathways as being associated with the brittle star early arm regeneration. Many of the genes identified in this study were not mentioned in previous reports, probably because of the differences in their analysis approach. Similarly, although the continuous expression pattern of genes associated with development, ECM and the cytoskeleton could be noticed, many of the common genes associated with these components and processes were not mentioned in this study.

Other than the above studies on *A. filiformis* arm regeneration*,* transcriptomic studies have been performed on two other brittle star species. One of these focused on arm regeneration of the Antarctic brittle star *O. victoriae* (Table 1) to determine if there were distinctive genes that cause slower regeneration rates in this species (Burns et al., 2013). Interestingly, regardless of the extensive regeneration time of *O. victoriae,* transcriptome profiles were like those previously reported for *A. filiformis,* with major representation of genes commonly found to be involved in regeneration. A recent study focused arm regeneration in another brittle star species *O. brevispina* (Table 1) (Mashanov et al., 2020). In this study*,* a total of 1,978 upregulated and 2,434 downregulated transcripts were identified in samples treated with a Notch pathway inhibitor. This study is further discussed in *Pharmacological Modulations*.

Taken together, the molecular studies of arm regeneration in brittle stars reinforce what has been seen at the cellular level, where common cellular events are taking place such as apoptosis, proliferation, migration, and differentiation. The differential expression of genes associated with development, remodeling, transcription, and metabolic activity provide the molecular effectors that underlie the initial events of the regeneration process.

#### Sea Cucumber Regeneration

In holothurians, the molecular aspects of regeneration have been studied in different species and in various organs (Table 2). Regardless of the differences in tissues and stages evaluated, GO terms and pathways analyses have yielded similar results. Some of the represented GO terms are metabolic processes, cellular signaling, binding processes, cell adhesion, and transcription/translation processes. Among the enriched pathways identified are ribosome, proteasome, development, signaling (e.g., *Notch, TGF-beta, Wnt*), and metabolic pathways. Collectively, these reveal the grand requirement of resources and mechanisms for the regeneration of the lost tissue. In the same way, the genes identified can be categorized in three main groups based on their involvement in specific processes: developmental, cytoskeletal and ECM component genes. Hence, they are accordingly discussed below, along with important differences between the studies.

While the focus of regeneration studies in holothurians has been the intestine, regeneration of other tissues and organs has also been investigated. For instance, the only transcriptome study on nerve regeneration of an echinoderm species was done on *H. glaberrima* (Mashanov et al., 2014)*.* In this model, the radial nerves are known to regenerate following transection (San Miguel-Ruiz et al., 2009). The cellular aspects of this regenerative process have been well described in a series of papers (San Miguel-Ruiz et al., 2009; Mashanov et al., 2013; Mashanov et al., 2017). In brief, it is known that fibers and cells migrate from both nerve stumps forming a bridge that eventually gives rise to a new nerve cord region that is scar-free. Regeneration occurs in about a month and radial glia-like cells play a key role as precursor cells for the new structure (Mashanov et al., 2013)*.* RNA-seq analyses of days 2-, 12-, and 20-post-injured radial nerve cords yielded a total of 4,023 upregulated and 3,257 downregulated transcripts. Functional analysis showed a high enrichment of transcripts involved with the ECM components and processes at all stages, when compared to uninjured nerve tissue. Comparatively, there was an upregulation of genes related to developmental processes at all analyzed time points, as has also been demonstrated for other tissues. At the earliest time points (2- and 12-days post injury) there was a high expression of genes involved in DNA synthesis and the cell cycle. Among the results of the study that should be highlighted are 1) the discovery of an increased expression of transposons that can be associated with the regeneration process (discussed extensively below) and 2) the finding that *Myc*, a Yamanaka factor (associated with induced cell plasticity) was upregulated in the early stages of radial nerve regeneration. The expression and role of *Myc* was the focus of other regeneration studies in this species (see below). In contrast, and surprisingly, *Bmi-1* pluripotency factor appeared to be downregulated. Furthermore, the study identified 11 transcription factors with potential roles in the control of genes during regeneration processes. Among these, the top differentially expressed were the serum response factor (*SRF*) (upregulated), involved in neuronal cell migration and axon guidance, and the zinc protein pleomorphic adenoma gene 1 (*PLAG1*) (downregulated). Many of the pathways and processes identified in these studies correlate with those described in the regeneration of other tissues and organs.

The study described above demonstrate the advantage of sea cucumbers as regeneration models for nerve cord regeneration. Beyond nerve regeneration, these model organisms can also regenerate internal organs, providing comparative studies on nerve regeneration and visceral organogenesis. Specifically, studies targeting the regeneration of their digestive tract, exemplify their suitability as regeneration model. The holothurian intestine comprises much of its digestive tract. This organ is similar in its histological structure to that of other metazoans and includes tissues that arise from the three different germinal layers: ectoderm, mesoderm, and endoderm. It is physically separated from most other tissues of the animal and can be easily isolated. More importantly, in many holothurians, the intestine is expelled from the animal in a process named “evisceration”. This is a natural process that can be induced in the lab by various methods. The new intestine regenerates from the tip of the mesentery to which the eviscerated intestine was attached, and the cellular events associated with the regeneration process have been well documented (Hyman, 1955; García-Arrarás and Greenberg, 2001; Mashanov and García-Arrarás, 2011; García-Arrarás et al., 2019; Quispe-Parra et al., (2021b)). These cellular events, except for some species-specific differences, appear to be shared by most species studied. The molecular aspects of intestinal regeneration have been studied in three holothurian species: *E. fraudatrix*, *A. japonicus,* and *H. glaberrima*, providing insights to regenerative processes that go beyond those that might be specific to holothurians.

The first large-scale molecular studies of intestinal regeneration performed in a holothurian were done in *H. glaberrima*, using expressed sequence tags (EST) and microarrays (Rojas-Cartagena et al., 2007; Ortiz-Pineda et al., 2009). These initial studies set the ground for many of the future molecular studies. The EST sequence profiling identified 5,173 differentially expressed sequences from intestinal rudiments of 3- and 7-days post evisceration (dpe) (Rojas-Cartagena et al., 2007). This provided one of the first clear views of the expression trends between regenerative stages, as libraries from each stage only shared 10% of the sequences, demonstrating a stage specific expression profile. For instance, at the earliest stage (3-dpe) one of the most represented transcripts was *serum amyloid A* (*SAA*) (Santiago et al., 2000; Santiago-Cardona et al., 2003). Conversely, at the 7-dpe stage there was a high representation of sequences corresponding to matrix metalloproteinases (*MMPs*), which are related to the ECM remodeling. Comparatively, *NF-kB* transcripts, which are related to inflammatory response, appeared to be downregulated at 7-dpe. All these findings coincide with the processes represented at this stage, including cell adhesion, proliferation and intracellular signaling. Other interesting transcripts were also identified as highly expressed at one or more stages, such as melanotransferrin (*Mtf*), *centaurin* and many other unknown transcripts. These unknown transcripts were part of some of the most represented sequences, as was one containing an EF-hand domain with high expression at 3-dpe. In regenerative studies of non-traditional model species, such as sea cucumbers, these uncharacterized transcripts are an important aspect as they could lead to potential key players in the process.

Custom microarrays based on previous EST sequences were done, profiling differential gene expression of 3-, 7-, and 14-dpe regenerating intestines, and compared to normal intestines (Ortiz-Pineda et al., 2009). As expected, this analysis yielded a higher percentage of characterized genes, compared to the first report. Therefore, it led to the identification of many genes associated with regenerative processes, such as cytoskeletal genes (i.e., *actins*), developmental genes (i.e., *Wnt* and *Hox*), and ECM-related genes, which were not identified in previous analyses. Gene expression profiles were analyzed with higher resolution, having a wider view of the expression profiles at different stages. For instance, developmental genes, such as specific *Hox* genes, showed upregulation at earlier stages (*Hox 9, 10,* and *12*), while others appeared upregulated at later stages (*Hox 5*). On the other hand, *Wnt14* showed continuous upregulation at all regenerating stages and *BMP-1* was only upregulated at 7-dpe. Many of the ECM related genes (i.e., *collagen, tenascin, laminin,* and *echinonectin*), showed upregulation at all regenerating stages, while the *MMP* genes returned to normal expression at 14-dpe. While many developmental and ECM related genes were upregulated at most regenerating stages, identified cytoskeletal genes showed the highest expression variability. Specifically, *alpha-tubulin-1 and -2, and actin-1* and *-2* were upregulated at all time points, whereas *gelsolin, myosin, and actin-3* were downregulated. Similarly, to the initial study, analyses resulted in numerous unknown genes that did not have significant homology with genes from public databases for other species. Many of these were differentially expressed primarily at 3- and 7-dpe. Hence, these two reports (Rojas-Cartagena et al., 2007; Ortiz-Pineda et al., 2009) were some of the first studies to demonstrate the potential of wide transcription profiling for the identification of novel genes that were not previously considered to be involved in tissue regeneration processes.

Following the initial high-throughput molecular studies on *H. glaberrima,* gene expression studies from *A. japonicus* using pyrosequencing technology were also reported (Sun et al., 2011). In contrast to experiments performed in *H. glaberrima*, the tissues assessed were pooled samples from 7-dpe regenerating intestines and 4-days regenerating body walls that were compared to normal intestine and body wall tissues. Therefore, rather than assessing tissue specific transcripts, they aimed to identify genes that might stand out in regenerating tissues. They identified 324 genes that were upregulated and 80 downregulated. The low number of differentially expressed transcripts is interesting, when considering that a total of 24,867 contigs were assembled. The identified genes were representatives of the common processes mentioned before, such as metabolic processes and translation regulation. Furthermore, numerous developmental transcripts with high regulation were identified including *frizzled* (*Wnt* pathway)*, Notch, Delta* (*Notch* pathway)*,* and *BMP* (*TGF-beta* pathway). They also found members of the development associated *kruppel-like family* (KLF) that were upregulated in the regenerative tissues. In another transcriptome study from this group, they found another member of this gene family (*KLF-6*) downregulated in early stages of regeneration of *A. japonicus* (Sun et al., 2013). Genes that form part of the ECM component were also identified as upregulated including *laminin*, *collagen*, and *tenascin* genes. Various cytoskeletal genes were also upregulated on the regenerative tissues, including multiple *actins* (i.e., *actin*, *actin-75*, *Actin-related protein 2–3*), *myosin* and *tubulin* genes. Different from prior studies, here they also identified epigenetic reprogramming genes, such as *Chromodomain-helicase-DNA-binding protein 5*, as differentially expressed. This study provides an overview of genes that may have a function in multiple regenerative processes and not necessarily in a specific tissue or at specific stages.

Many of these genes were also identified in a later study where the same research group performed RNA-seq on regenerating intestines at different stages (3-, 7-, 14- and 21-dpe) (Sun et al., 2013). Different from the pooled data analysis of the first study, here they compared each individual stage to non-eviscerated intestinal tissue. The study yielded an improved characterization of differentially expressed genes and their possible involvement in specific pathways and processes in *A. japonicus*. In general, results coincided with those found in the initial studies of *H. glaberrima;* many of the similar expression trends were those of developmental, cytoskeleton and ECM related family genes. As seen before, their results showed that the extent of the differential expression in earlier stages was higher compared to those at later regenerative stages. The authors went in depth about the differentially expressed genes and enriched processes/pathways at each individual stage, demonstrating the transcription dynamics of the regrowth of the intestine. These dynamic changes are exemplified by focusing on those genes previously recognized as important for regeneration. Summarizing for the developmental genes, *Wnt4 and Wnt6* reached their peak expression at 7-dpe and slowly decreased as regeneration progressed. A similar expression pattern to that of *Wnt* genes*,* was seen in *Hox1* and *Hox3* with maximum expression peaks in the 3-dpe regenerating intestine. On the other hand, and different to that found before, *KLF-6* showed downregulation at 3-, 7- and 14-dpe, but upregulation at 21-dpe. The expression patterns of genes of ECM components showed diverse regulation patterns within specific families. For example, *MMP-1* and *-12* had high expression at 7- and 14-dpe, and *MMP-16* appeared upregulated at 3- and 7-dpe, while *MMP-14* appeared downregulated at 3- and 7-dpe. Cytoskeletal genes showed specific expression profiles with alpha-*tubulin*, beta-*tubulin*, and *actin* showing high expression, while *myosin*, *gelsolin* and gamma-*tubulin* appeared downregulated. Many of the same GO terms that had been seen so far prevailed. However, no significantly enriched GO terms were identified at 21-dpe, perhaps due to the low number of differentially expressed transcripts at this stage. Therefore, this decrease of transcription activity at this stage compared to the earliest, demonstrate that an increment in cellular activity is needed once regeneration is initiated. Further, compared to other studies, in their pathway analysis they showed not only those enriched by upregulated genes (e.g., *Notch* signaling pathway, ribosome, and spliceosome), but also depicted those highly represented by downregulated genes (e.g., digestion and absorption of vitamin fats and carbohydrates, Renin-angiotensin system).

In this study they compared 3-dpe relative to 7-dpe. This comparison is important, as the tissues from these stages are more similar between them than they are to normal intestine, providing for more resolution in determining gene differential expression. The most enriched processes from this comparison were those related to serine family amino acid and polyol metabolism. However, the only pathway that was unique in this comparison was the Glycine, serine, and threonine metabolism pathway. Moreover, among the top significantly differentially expressed genes were calcium activated chloride channel and solute carrier family 5, which were upregulated, and cellular retinol-binding protein type 1b and annexin A7 that were downregulated in 3-dpe.

A third holothurian species, *E. fraudatrix,* has been studied using high-throughput transcriptomic analysis. This study was centered on the transdifferentiation mechanism of mesodermal cells as part of the regeneration process, since in this species, the coelomic epithelia of the anterior regenerating intestine gives rise to the luminal epithelia (Boyko et al., 2020)*.* Rather than describing the whole regeneration process, the study provides an insight into the transcription factors that are potentially involved in transdifferentiation during intestinal regeneration. For this study, the profiling was done focusing on the expression at 5-7-dpe. From their comparisons, 11 upregulated transcription factors at 5-7-dpe were identified as potential regulators of transdifferentiation. These included the early growth response 1 (*EGR1*), E74-like ETS transcription factor (*ELF*), GATA binding protein 3 (*GATA3*), inhibitor of DNA binding 2 (*ID2*), *KLF1/2/4*, musculin (*MSC*), polycomb group ring finger 2 (*PCGF2*), *PRDM9*, snail family transcriptional repressor 2 (*SNAI2*), T-Box transcription factor 20 (*TBX20*), and transcription factor 24 (*TCF24*). These have been reported to be mostly involved in development, cell reprogramming, cell proliferation, and cell differentiation. Importantly, even though the study mainly focused on the 11 transcription factors above, other genes such as *Sox17*, also appeared to be highly expressed at the first stage of regeneration. In fact, *Sox17* is one of the identified genes in the transcriptome profile of the polian vesicles of eviscerated sea cucumbers, where production of coelomocytes occurs (Shi et al., 2020). Certainly, although the analysis of *E. fraudatrix* intestinal regeneration gene profiles had a specific focus on transdifferentiation processes, the identified transcription factors might also represent additional processes occurring simultaneously.

The most recent addition to the transcriptomic studies of holothurian intestinal regeneration is an in-depth study using RNA-seq on early stages of intestinal regeneration of *H. glaberrima* (Quispe-Parra et al., 2021a). The study focuses on 1-dpe and 3-dpe, the time when the cellular dedifferentiation process begins. This was the first transcriptomic analysis on stages earlier than 3-dpe and the first in comparing the early regenerating intestine to the mesentery of non-regenerative animals, instead of to normal intestine. The study yielded the differential expression of 8,460 transcripts at 1-dpe and 8,216 at 3-dpe. Both stages shared a total of 3,884 differentially expressed transcripts. These results clearly show major differences from those yielded in *E. fraudatrix, A. japonicus,* and even from previous studies from our group in *H. glaberrima*. These differences were mainly seen in the expression profiles of certain genes and the identification of new gene candidates. Among the top upregulated genes at 1-dpe were *actin, serine/threonine-protein kinase NLK, translation elongation factor 2 (TEF2), and elongation factor 1 alpha (EEF1A).* Among the top downregulated genes were the pancreatic lipase-related protein 2 (*PNLIPRP2*), sodium-coupled monocarboxylate transporter 1, histidine ammonia-lyase, caudal homeobox protein (*CDX-2*), and *aquaporin*-8. Some of these downregulated genes maintain their downregulation at 3-dpe (i.e., *CDX-2*, sodium-coupled monocarboxylate transporter 1, and *aquaporin*-8). At 3-dpe some of the top regulated genes were *ubiquitin, actin* and *Wnt6*. Interestingly, many of the genes identified at both stages were not among the top differentially expressed genes in previous intestinal regeneration studies. This could be mainly attributed to differences in the type of tissues that were compared, different stages or the different analyses performed. Out of the mentioned upregulated genes at 3-dpe, only *actin* and *Wnt6* were also identified as upregulated in *A. japonicus.* One of the highlights of this study was the description of the differential expression of transcription factors. Among those transcription factors identified were *Myc, KLF13, and Sox4*, which showed constant higher expression, while others, such as *CDX1*, appeared with constant low expression. Moreover, genes involved in transcription appeared to be upregulated at 1- and 3-dpe (*TAF1A*, *MYBBP1A*, *PWP1*, and *EEF1A*).

We should also mention transcriptome analyses performed in other holothurian tissues that are related, to a certain extent, to the evisceration and regeneration processes. One of the most recent, is the transcriptome profiling of *A. japonicus* polian vesicles (Shi et al., 2020). Polian vesicles are known to be part of the inflammatory responses and have been suggested to be a site of origin of coelomocytes, the echinoderm immune cells. Polian vesicles remain within the animals after autotomy of other internal organs and are thought to be involved in coelomocyte recovery after evisceration. The study performed RNA-seq of the polian vesicles 6 h post-evisceration. Results showed that differentially expressed transcripts were part of development and signaling pathways, such as are *Wnt, TGF-beta* and Endocytosis pathways, all known to be involved with cellular proliferation and differentiation. Hence, data strongly suggests that following evisceration, the polian vesicle goes through a distinct transcriptome activity, possibly due to the great production of coelomocytes, strengthening their proposed role in the inflammatory response of sea cucumbers upon evisceration.

Another recent study performed transcriptomic profiling of genes expressed during the initiation of fission of the holothurian *Cladolabes schmeltzii* (Dolmatov et al., 2018)*.* Fission is a form of asexual reproduction, during which sea cucumbers constrict around the middle region due to the changes in the connective tissue of the body wall. In this study the focus was on ECM-related genes, as this change in the strength of the connective tissue would not be possible without ECM remodeling. Many genes that form part of structural proteins of the ECM and proteases were identified in animals undergoing fission*.* Furthermore, there were development-associated genes that were expressed*.* Importantly, they also identified numerous transcription factors (26 in total) that were activated during fission. This high number of transcription factors is thought to be due to the regeneration processes that begin right after the division of the animal. There are specific transcripts that support this as they have been identified to be involved in the generation of the digestive system during development, such as genes from the *Sox and GATA* family. Among the tissues sampled for the analyses, they included the remnant mesentery, and the same genes were also identified in the latest transcriptome profiling of the sea cucumber *H. glaberrima* by Quispe-Parra et al., 2021a.

The evisceration mechanisms of sea cucumbers, which trigger the subsequent intestinal regeneration, have also been subjected to transcription profiling (Ding et al., 2019). In *A. japonicus*, the tissues include the nerve ring, including the calcareous structure, as well as the associate muscles of animals that were 1) eviscerating animals, 2) 3-h post evisceration and 3) non-induced to eviscerate (normal). The analysis yielded differentially expressed genes related to response to stimulation, muscle contraction, metabolism, ECM and secretion of neurotransmitters. In the same way, there were also genes related to the regeneration process identified mostly at the 3-h post evisceration, such *SAA* and *MMPs*. These studies provide a broader point of view of the interactions among body components, where processes taking place before or after regeneration can have an effect on the regeneration of other organs.

#### Transposable Elements

As mentioned earlier, transcriptome studies provide the opportunity to discover genes that have not been previously considered to be involved in a particular process. Such is the case with the finding that a large number of transposable elements (TEs), or transposons, were differentially expressed at different stages of radial nerve regeneration in *H. glaberrima* (Mashanov et al., 2012c). TEs are genomic DNA sequences that are able to “jump” from one position to another by copying themselves and inserting the copy DNA into a new location or by excising themselves from the genome and reinserting into a new location. As genomic and molecular studies continue to evolve, TEs have started to become more relevant and some of their roles have become evident in various species. Therefore, we consider that they are part of the advancements made towards identifying the molecular regulators of regeneration.

The first time TEs were associated with regeneration was in a study by our laboratory that found elements of the *Gypsy* and *BEL* LTR families differentially expressed at different stages of radial nerve regeneration on *H. glaberrima* (Mashanov et al., 2012c). It was further reported that two *Gypsy* retrotransposons were also expressed at various stages of intestinal regeneration (Mashanov et al., 2012b). In a similar way, retrotransposons accounted for about 33% of genes identified during fission initiation in *C. schmeltzii* (Dolmatov et al., 2018). In other regeneration models, the non-LTR LINE-1 was shown to be activated during limb regeneration of the salamander (Zhu et al., 2012). Similarly, a study on the Iberian ribbed newt, *Pleurodeles waltl,* also showed that specific TEs, including *Gypsy* elements were upregulated during limb regeneration and that these appear to have been expanded in this species genome (Elewa et al., 2017). Similarly, another study on lungfish tail regeneration, the authors found 16 TEs upregulated in the regenerating tail blastema (Verissimo et al., 2020). Thus, multiple studies on TE sequences demonstrate their expression in a dynamic manner, suggesting a functional role in the regeneration processes.

Other than the identified retrotransposons expressed during regeneration and fission of sea cucumbers mentioned there are no further studies addressing their role during tissue regeneration or other processes in echinoderms. Although there is a limited knowledge about retrotransposons in invertebrate species with advanced regenerative capacities, current data suggests that their abundant representation in the genome of these species (Flowers and Crews, 2018; Nowoshilow et al., 2018; Biryukov et al., 2020) and their upregulation after injury might be correlated to a species regenerative capacity. The degree to which these sequences are involved in this process is still unexplored, but as more genomic data is generated for distinct echinoderm species, we will be able to answer questions about these elements that were previously difficult to approach.

#### Genes From Genomic Studies

In the last decades several echinoderm genomes have been assembled, including that of *S. purpuratus, L. variegatus, Parastichopus parvimensi, Anchaster planci,* and *P. miniata,* among many others, which have been addressed in other publications and databases, such as echinobase (Cameron et al., 2015; Cary et al., 2018). However, these genomes have not been explored or discussed in relation to the animal’s regeneration capacity. Here we focus briefly only on the published genome of *A. japonicus*, which contains information discussed by the authors as to be relevant to its regeneration potential.

In 2017, the genome of the sea cucumber *A. japonicus* was sequenced (Zhang et al., 2017). Among the findings that were highlighted were the presence of two protein families that appeared to be significantly expanded when compared to other animal species. One of these families encoded a group of prostatic secretory proteins of 94 amino acids (PSP94)-like genes while the other was a family of fibrinogen-related protein (FREP) genes. The presence of these genes in a highly regenerative species, led the authors to posit that these proteins played a role in the organism’s intestinal regeneration process. Gene expression studies showed that these two gene families were upregulated in early regenerating intestines. Many other genes were studied in the regenerative process that follows evisceration in *A. japonicus*, however, the use of normal intestinal tissues as controls, particularly for the early regenerative stages makes it difficult to determine the relevance of the obtained results. As highlighted by (Quispe-Parra et al., 2021a), results obtained when comparing normal intestine to 3-dpe rudiments might correspond more to cell type-specific expression than to regeneration-associated expression.

### Category 3—Functional Studies

The experiments described in the preceding section provide an important list of possible candidate genes that could control or modulate the regenerative process. However, the results are only correlative and there is no concrete proof that the listed genes are involved in regeneration. More so when we realize the multiplicity of processes, other than regeneration, that are taking place following evisceration, including activation of the immune system to deal with incoming pathogens, wound healing responses in the injured tissues and metabolic changes due to the loss of the main digestive organ, among others. Therefore, the need to perform experiments that probe the identified genes to determine their function during the regeneration process.

### Pharmacological Modulations

Most studies performed to determine the role of particular molecules or pathways during echinoderm regeneration have been done using specific drugs that inhibit enzymes, receptors or components of a signal pathway. For most of them, previous experiments had shown that the molecules of interest were present in the tissues or organs studied and that they were over-expressed during the regenerative process, thus providing the rationale for their functional analyses. Pharmacological studies were done, in most cases, by treating the animals with a drug or chemical that interfere with the molecule function while undergoing regeneration and the results were compared to those of animals treated with the vehicle. While several species/pathways have been targeted using this pharmacological strategy (see below), its strength is demonstrated in two model systems: in the brittle star *A. filiformis* to study the role of fibroblast growth factors (FGF) during arm regeneration and in the sea cucumber *H. glaberrima* to analyze the role of the Wnt-βcatenin pathway during intestinal regeneration.

#### MMPs

One of the first pharmacological studies performed, targeted the matrix metalloproteinases (MMPs) as a way of studying their function in the extracellular matrix remodeling shown to take place during intestinal regeneration in *H. glaberrima* (Quiñones et al., 2002). In these experiments MMP activity was shown to increase during intestinal regeneration, and this took place concomitantly with collagen degradation. To study their possible function, three MMP inhibitors were used [1,10-phenanthroline, N-CBZ-Pro-Leu-Gly hydroxamate and p-aminobenzoyl Gly-Pro-D-Leu-D-Ala hydroxamate. All three caused a decrease in the size of the rudiment, suggesting that MMP activity is necessary for normal regeneration of the intestine. Similar results were obtained with other drugs in other species. (Dolmatov et al., 2019). treated a different species of sea cucumbers (*E. fraudatrix*) with a different MMP inhibitor (GM6001) after transecting the ambulacrum (body wall area including radial nerve, hemal vessel, water vascular canal and muscle band]. Their results showed not only a possible role for MMPs in regeneration, but also the importance of the timing of enzymatic activity; animals that received the drug 3 days after injury could not undergo wound healing and died, while animals that received the drug later (7-days) survived, and slowly regenerated.

#### RGD

Intracelomic injections of arginine-glycine-aspartate (RGD)-containing peptides were used to study the role of the ECM during regeneration (Cabrera‐Serrano and García‐Arrarás, 2004). These peptides blocked the association between cellular integrins and ECM molecules, and caused a delay in the regeneration process, as determined by a smaller rudiment size and a decrease in the ECM remodeling. Moreover, the presence of the RGD-containing peptides appeared to interfere with cellular migrations within the connective tissue of the mesentery.

#### Retinoic Acid

Components of the retinoic acid (RA) signaling pathway were identified in the digestive tract of the sea cucumber *H. glaberrima*, and some of these were shown to be differentially expressed during regeneration (Viera-Vera and García-Arrarás, 2018; Viera-Vera and García-Arrarás, 2019). To test the function of the RA signaling pathway, an inhibitor (Citral) against one of the RA synthesizing enzymes and an antagonist (LE135) of the retinoic acid receptor were administered to animals undergoing intestinal regeneration. Both drugs caused a decrease in the size of the regenerating rudiment and a significant reduction in cell division and cell dedifferentiation. Thus, suggesting that the RA signaling pathway has a role in the modulation of the cellular processes that are important for the regeneration of the intestine.

#### Proteasome

Components of the proteasome were found to be overexpressed during the early stages of intestinal regeneration (Pasten et al., 2012a). To study the role of the proteasome during this process, intracoelomic injections of MG132, E64d and TPCK were done in regenerating animals (Pasten et al., 2012a; Pasten et al., 2012b). These drugs disrupt the function of the proteasome through different mechanisms: MG132 is an inhibitor of the chymotrypsin and PGPH activity, E64d is an inhibitor of calpains and some cathepsins and TPCK is an inhibitor of serine proteases. TPCK showed no effect at the dose used, while MG132 and E64d treatments showed several effects on the regenerative cellular processes. Both drugs reduced cellular proliferation in the intestinal rudiment, however MG132 treated animals showed a reduction in the size of the rudiment while E64d treated animals showed a delay in the degradation of collagen. These types of experiments serve to separate cellular processes and the signals that might be modulating them during the regenerative response.

#### Notch

Pharmacological inhibition of the Notch pathway was done in sea urchins using DAPT (Reinardy et al., 2015). In these experiments both spines and tube feet regrowth were inhibited by intracelomic doses of DAPT. Moreover, animals treated with the Notch inhibitor showed a decrease in the expression of Notch target genes: hey, gataC and hes (Reinardy et al., 2015). DAPT was also tested during arm regeneration of the brittle star *Ophioderma brevispina* (Mashanov et al., 2020). Here also, inhibition of the Notch pathway significantly reduced the regrowth of the regenerating arm, suggesting that Notch plays a role in echinoderm regenerative processes particularly on those related to the regeneration of appendages. The authors also performed a comparative transcriptomic study between animals regenerating their arms in the presence and absence of the Notch pathway inhibitor. Differential expression of genes modulated by Notch pathway inhibition extended beyond the classical Notch target genes and effects were observed in many other signaling pathways that control cell proliferation, survival, apoptosis, differentiation and immunity, thus highlighting the interconnection of multiple pathways that takes place during the regenerative process. Their analysis of the data led the authors to propose that one gene in particular, Neuralized1-might be playing an important role in the process.

#### FGF

The effect of FGF signaling inhibition was studied in the regenerating arm of the brittle star *A. filiformis* (Czarkwiani et al., 2021). These studies focused on perturbations in skeletogenesis and were accompanied by extensive analyses of gene expression that served as molecular markers. The drug used was SU5402, a specific inhibitor of FGF receptors that competes with ATP for the tyrosine kinase catalytic domain. The authors exploited one of the main advantages of using echinoderm models; the possibility of comparing regenerative responses to embryonic development. Thus, they compared the gene expression profile of skeletogenesis during arm regeneration to that of the initial formation of the skeleton in the brittle star embryo. They further probed the effect of the FGF receptor inhibitor in both regenerating adult and developing embryonic stages. Embryos treated with the FGF receptor inhibitor failed to form the skeletal spicules needed to form the larval skeleton. Similarly, FGF receptor inhibition in adults prevented skeletal spicule formation in the regenerating arms (Figure 3). Interestingly, cell proliferation continued in the presence of the drug, once again highlighting the independence of certain cellular processes.

In the same report, VEGF was also studied in embryos and adults, by blocking its receptor with the drug axitinib. The response was a much milder effect when compared with blockade of the FGF receptor. The authors suggest that VEGF is not strictly required for skeleton formation but might be needed for establishing the patterning of the spicules. However, these results draw attention to one of the main problems encountered by those doing experiments in invertebrate or non-mammalian model systems: the possibility that the effect of a drug that has been tested on vertebrates (or more specifically on mammals) differs in other animal groups due to variations in the structure of the drug target (i.e. protein sequence of receptors or enzymes); in the present case in the VEGF receptor that is targeted by the axitinib.

### Gene Expression Modulation (Knockdown)

#### Myc

The first report of gene knockdown in adult echinoderms was performed using the regenerating radial nerve cord of holothurians as a model system. The target gene was *Myc*, a gene that had been previously shown to be overexpressed in the radial nerve cord cells following cord transection (Mashanov et al., 2015a). A dsRNAi protocol was developed where the dsRNA was electroporated at the same time that the cord was transected (Mashanov et al., 2015b). The subsequent decrease in *Myc* mRNA levels (and the expected decrease in Myc protein levels) was accompanied by a decrease in the dedifferentiation of radial glia and a decrease in cellular apoptosis. These results strongly position *Myc* as one of the key genes controlling the initial events in the nervous system regenerative response. At the same time the experiments established a path on how the function of other candidate genes could be tested.

#### Wnt

The most complete analysis on the molecular basis of regenerative processes in echinoderms is probably the study of the *Wnt* signaling system in intestinal regeneration in holothurians. This pathway is present in all metazoan and involves the activation of membrane receptors by soluble proteins of the *Wnt* family that are usually released by neighboring cells (Nusse and Clevers, 2017). Among the signaling pathways modulated by *Wnt* is the canonical or Wnt/β-catenin dependent pathway. This pathway has been associated with multiple developmental and regenerative processes (Whyte et al., 2012). For example, *Wnt* has been associated with *Hydra* apical regeneration (Vogg et al., 2019), the establishment of axial polarity in regenerating Planaria (Almuedo-Castillo et al., 2012) and tail regeneration in zebrafish and in *Xenopus* tadpole (Stoick-Cooper et al., 2007; Lin and Slack, 2008) among others. Moreover, the canonical *Wnt*/*β-catenin* pathway is known to play a key role in the maintenance and regeneration of the luminal stem cells of the vertebrate intestine (Cordero and Sansom 2012; Kretzschmar and Clevers 2017).

The study of the *Wnt* signaling pathway genes in holothurian intestinal regeneration encompasses all previously described categories of gene molecular studies. First, mRNA for *Wnt* genes and/or some of their target molecules have been identified within the regenerating intestinal rudiment in at least three species of holothurians (Mashanov et al., 2012a; Sun et al., 2013; Girich et al., 2017; Li et al., 2017; Yuan et al., 2019). Moreover, the expression of some of these molecules has been shown to differ significantly at some stage of intestinal regeneration. For example, *Wnt9* was shown to increase its expression during *H. glaberrima* intestinal regeneration (Ortiz-Pineda et al., 2009) and transcripts were detected within the regenerating intestinal rudiment (Mashanov et al., 2012a). *Wnt6* and *WntA* were detected in intestinal tissues of *A. japonicus,* and their expression shown to vary depending on the stage of intestinal regeneration (Sun et al., 2013; Li et al., 2017). Finally, four different *Wnt* (A, 4, 6 and 16) were detected in regenerating internal organs of *E. fraudatrix* and each shown to have a distinct expression profile during the regeneration process (Girich et al., 2017).

Further studies in *A. japonicus* showed that *Wnt7* and two upstream genes in the *Wnt* signaling (*Fz7* and *Dvl*) were also differentially expressed during intestinal regeneration and provided a positive selection analysis that strengthened the importance of this signaling pathway in intestinal regeneration (Yuan et al., 2019). Analysis of conservation demonstrated the positive selection of *Wnt* signaling pathway genes among echinoderms. Furthermore, the high expression of upstream genes (*Wnt7* and *Frizzled7*) at early intestine regeneration and downstream genes (*Myc*) at advanced stages strongly suggested an early activation of the pathway upon the initiation of regeneration. These authors also performed some experiments with a pharmacological inhibitor of the *Wnt* pathway and RNAi using dsRNA, and although the results confirm a possible role of *Wnt* on the regeneration process, the paucity of technical details and controls weaken the reliability of the findings.

Thus, the *Wnt* pathway genes fulfill two of the requirements for a potential role (or roles) in the intestinal regeneration process: 1) genes associated with the pathway are expressed in cells of the regenerating intestinal rudiment and 2) some of these genes show differential expression associated with specific stages of the regeneration process. Pharmacological studies *in vivo* and *in vitro*, provide additional (and much stronger) evidence for an active role. In *H. glaberrima*, injections of *Wnt* pathway inhibitors and activators into the coelomic cavity of regenerating animals showed significant changes in intestinal rudiment and in some cellular processes (Bello et al., 2020) (Figure 3). *Wnt* pathway inhibitors were shown to cause a decrease in the size of the regenerating rudiment, while activators showed an increase in the size of the structure. *In vivo* pharmacological studies, however, are prone to possible side effects, particularly when dealing with drugs that have a wide action spectrum, or that affect multiple processes. The research was then advanced by establishing an *in vitro* intestinal explant setup where certain cellular events associated with regeneration could be studied (Bello et al., 2020). Using this model system, multiple *Wnt* pathway modulating drugs were tested. The overall conclusion of the experiments was that the canonical *Wnt*/*β-catenin* pathway was responsible for the increase in cell proliferation associated with intestinal regeneration, but it had no effect on two other cellular events: muscle cell dedifferentiation nor apoptosis. Further studies suggested that dedifferentiation was under the control of a *GSK3*, *Wnt*-independent pathway that remained unidentified.

Although, experiments with pharmacological drugs are a step in the right direction, they remained, in many ways, inconclusive. This is due, as explained above, to the possibility of drug effects on other cells, or on other cell processes that are occurring concomitantly with regeneration. In addition, even in the best cases, the specificity of the drugs has to be questioned. Some of these drugs might modulate not only one gene or gene product in a signaling pathway but might modulate all members of a gene family. For example, in holothurians, at least four different *Wnt* genes have been detected during intestinal regeneration (Girich et al., 2017). When animals are treated with an inhibitor of the Wnt pathway, it can be modulating the expression (and thus, the role) of all these genes simultaneously.

In other animal models, the ultimate proof to define the role of a particular gene is to knock-out or knock-down the gene in question and then determine the effect of the genetic manipulation on the process being studied. Although many gene modulation techniques are available for echinoderm embryonic studies, their application for studies in adult echinoderms is a serious limitation to the regeneration field. Thus, the importance of the recent development in our lab of a dsRNAi method to knock-down specific gene expression in regenerating intestinal explants (Alicea-Delgado et al., 2021). This methodology was applied to the study of the Wnt pathway during regeneration by targeting *β-catenin*, a key molecule in the intracellular Wnt signaling pathway. The levels of *β-catenin* mRNA in the regenerating intestinal explant were knocked-down by the electroporation of ds-*β-catenin* RNA (Alicea-Delgado and García-Arrarás, 2021). Concomitant with the decrease in *β-catenin* mRNA (and therefore on the activity of the *Wnt*/*β-catenin* pathway) a ∼50% decrease in cell division was observed. However, no effect on apoptosis or cell dedifferentiation occurred. These results coincide with those obtained with the pharmacological treatments (Bello et al., 2020). The use of complementary techniques and the similarity in results provide reassuring evidence, showing that cell proliferation during intestinal regeneration in holothurians is under the control of the *Wnt*/*β-catenin* signaling pathway. More importantly, our results indicate that we now have the tools to explore more in depth, the molecular basis of intestinal regeneration in holothurians.

## Conclusion

Echinoderm regeneration studies have advanced in recent years, and will become more numerous, thanks to the accessibility and increase of genomic and molecular data. However, this review shows the areas where more information is needed or where particular considerations must be taken for the data to be used for comparative studies.

On the issue of data availability, we have documented that most of the existing data focusing on the molecular base of echinoderm regeneration has been obtained from species of two echinoderm classes, holothuroids and ophiuroids. This makes further exploratory molecular studies on crinoids, echinoids, and asteroids essential in order to obtain a broader overview of the molecules that act during regeneration. Equally important is the opportunity presented by the recent studies on sea urchin and sea star larval regeneration that could allow the identification of molecular processes common to larval and adult regeneration. Or otherwise, identifying genes that might be activated specifically for regeneration purposes, and not development.

This review also highlights one of the main drawbacks of current available data: the heterogeneity of the tissues and stages used to perform gene expression studies within species. For instance, in ophiuroids, studies differ on the regions being dissected and on the description of regeneration stages with some focusing on differentiation index and other on days post amputation. Similarly in holothuroids, studies have questioned the use of adult normal intestines as being the appropriate comparisons for early regeneration intestines to determine differential gene expression due to dissimilarities in tissue layer composition. Therefore, there is a need of standardizing regeneration studies that could be achieved as more information on the process continues to be gathered.

Moreover, there is also need for more exploratory analyses, and therefore well-curated genomes and transcriptomes. So far data analysis and candidate genes assessment have been focused on specific gene groups—developmental, ECM, and cytoskeletal. From these groups most of studies have aimed to assess developmental genes, as they appear to be co-opted to participate in the regeneration of the lost tissues and organs*.* Notwithstanding, there is widespread ground to cover to clearly understand the molecular underpinnings of regeneration. Studies so far (and therefore this review) have been limited to exposing genes that are easily identifiable by database mapping, but very few have made efforts towards new gene discoveries. However, there are also numerous unknown transcripts among the top differentially expressed genes, which could be key molecules to the whole process. This displays the need of further characterized genomic data of diverse species. Certainly, the vast amount of echinoderm genomes and extensive transcriptome data exerts the possibility of identifying unknown echinoderm-specific orthologs and novel regulatory regions that could be crucial to prompt the regrowth of the lost tissue or organ.

Currently, there are numerous genomes and transcriptomes of echinoderm species with great regeneration potential that are waiting to be analyzed. As new genes are added to the growing list of “candidate” genes involved in echinoderm regeneration, new methods and molecular tools will need to be developed to determine gene function. This is probably the limiting factor, at present, to advance the molecular analyses of regeneration. The possibility of genetic studies, developing CRISPR-Cas methods and other techniques that are commonly used in other model systems will be a huge advance for echinoderm regeneration studies.

In summary, the great number of macromolecular studies currently available or in process will continue to provide new information on the molecular events associated with echinoderm regeneration. As new data becomes available, the importance of platforms such as echinobase where the echinoderm community can find reliable data and analyses, will continue to grow. This will provide the basis for comparative analyses establishing unique molecular characteristics that might be responsible for the amazing regenerative processes observed in echinoderms.
